# Processing of spatial sounds in human auditory cortex during visual, discrimination and 2-back tasks

**DOI:** 10.3389/fnins.2014.00220

**Published:** 2014-07-28

**Authors:** Teemu Rinne, Heidi Ala-Salomäki, G. Christopher Stecker, Jukka Pätynen, Tapio Lokki

**Affiliations:** ^1^Institute of Behavioural Sciences, University of HelsinkiHelsinki, Finland; ^2^Advanced Magnetic Imaging Centre, Aalto University School of ScienceEspoo, Finland; ^3^Department of Hearing and Speech Sciences, Vanderbilt University Medical CenterNashville, TN, USA; ^4^Department of Media Technology, Aalto University School of ScienceEspoo, Finland

**Keywords:** auditory cortex, spatial processing, fMRI

## Abstract

Previous imaging studies on the brain mechanisms of spatial hearing have mainly focused on sounds varying in the horizontal plane. In this study, we compared activations in human auditory cortex (AC) and adjacent inferior parietal lobule (IPL) to sounds varying in horizontal location, distance, or space (i.e., different rooms). In order to investigate both stimulus-dependent and task-dependent activations, these sounds were presented during visual discrimination, auditory discrimination, and auditory 2-back memory tasks. Consistent with previous studies, activations in AC were modulated by the auditory tasks. During both auditory and visual tasks, activations in AC were stronger to sounds varying in horizontal location than along other feature dimensions. However, in IPL, this enhancement was detected only during auditory tasks. Based on these results, we argue that IPL is not primarily involved in stimulus-level spatial analysis but that it may represent such information for more general processing when relevant to an active auditory task.

## Introduction

The spatial location of a sound source is not directly mapped along the sensory epithelium but must be computed based on various binaural cues. These cues result from differences in the level and arrival time of sound at the two ears, spectral cues resulting from direction-dependent filtering of sound by the head, outer ears and torso, overall intensity, and echoes in a reverberant environment. Binaural cues, in particular, are important for localizing the sound source in horizontal plane. Received sound intensity is the main distance cue as sounds are generally louder from a close than distant source. Reverberation and its level related to the direct sound are helpful as complementary distance cues and provide information on the characteristics of the environment (e.g., size of the room; Zahorik et al., 2005). While numerous brain imaging studies have investigated processing of sounds varying in horizontal plane, processing of distance and reverberation have received less attention (but see e.g., Kopčo et al., 2012). In the present study, we compared activations in human auditory cortex (AC; Woods et al., 2010) to sounds emitted by sources at different horizontal locations, at different distances, or in different rooms. As AC operations are strongly modulated by task, the sounds were presented during a visual, auditory discrimination, and auditory 2-back memory tasks. We also used naturalistic and carefully controlled sounds recorded in realistic acoustical spaces, as the different spatial cues are not processed independent of each other in natural listening conditions.

Spatial processing is central to current theoretical brain-level models of the human auditory system. In the prevailing models, human AC consists of anatomically and functionally separate fields organized in parallel processing streams. One prominent theory suggests that areas within a posterior stream, projecting from the posterior superior temporal gyrus (STG) to the inferior parietal lobule (IPL), are important for spatial processing (Rauschecker and Tian, 2000; Rauschecker and Scott, 2009; Recanzone and Cohen, 2010). This idea is supported by human fMRI studies reporting enhanced activations in these areas associated with processing of sounds with sources varying in the horizontal plane (Warren and Griffiths, 2003; Barrett and Hall, 2006; Alain et al., 2008; Kopčo et al., 2012). In particular, an area of the superior temporal plane posterior to Heschl's gyrus (HG), the planum temporale (PT), has been implicated in spatial processing. However, it is not clear whether modulation of activation within the posterior stream reflects spatial processing as such, or some other function such as stream segregation (e.g., Griffiths and Warren, 2002; Alain et al., 2008; Smith et al., 2010; Hickok and Saberi, 2012).

In addition to physical features of stimuli, AC activations are also strongly modulated by the characteristics of the task. For example, Rinne et al. (2012) compared AC activations to spatially varying sounds presented during spatial discrimination and spatial n-back memory tasks. They found activation enhancements in anterior AC associated with spatial discrimination, whereas spatial n-back memory task enhanced activations in posterior STG and IPL. However, the functional significance of such task-dependent modulations is not well understood. Further, it is not known whether feature specific and task dependent effects are independent of each other. An intriguing possibility is that the anterior and posterior processing streams are task defined rather than feature defined (Hickok and Saberi, 2012).

In the present study, we compared AC activations to sounds varying in distance (spatial distance, SD; near, middle or far), location in horizontal plane (spatial location, SL; left, middle or right), or that were emitted in different rooms (spatial room, SR; small, medium, large). These sounds were presented in separate blocks in which subjects performed a visual discrimination task, an auditory spatial discrimination task, or an auditory spatial 2-back memory task. We hypothesized that comparisons of activations to SD, SL, and SR sounds presented during the visual task (i.e., absent auditory attention) would reveal stimulus-dependent activation differences and similarities associated with processing of these different spatial features. Further, we expected that comparison of activations across visual, auditory discrimination and auditory 2-back tasks would reveal task-dependent effects. We also investigated whether activations to spatial sounds in PT and IPL differ in the three task conditions. If PT and IPL belong to same feature-specific processing stream, then they should show similar relative sensitivity to SD, SL, and SR sounds during all tasks.

## Materials and methods

### Subjects

Eighteen healthy subjects (ages 22–41, mean 27.8 years, 8 women, no known hearing deficits, 17 right handed) participated after providing written informed consent. The study protocol was approved by the Ethical Committee of the Institute of the Behavioural Sciences, University of Helsinki. Data from one subject were rejected due to inaccurate coregistration of functional and anatomical images.

### Stimuli

Spatialized sounds were generated by convolving a brief sound (a dry recording in an anechoic chamber of a snare-drum hit, i.e., the recording had no spatial cues) with impulse responses that combined the reverberant features of real rooms with the directional characteristics of a sound received binaurally by a human listener (facing the sound source in the room). Room impulse responses were first recorded in real spaces using a two-way loudspeaker as a sound source and a six-microphone array as a receiver. The responses were processed with spatial decomposition method (Tervo et al., 2013) to reconstruct a virtual 24-channel 3D loudspeaker setup. The loudspeaker responses were next convolved with head-related impulse responses measured from a generic human subject and corresponding to the directions of the virtual loudspeakers. The product of this two-step process is equivalent to direct recording of binaural impulse responses with a dummy-head, but additionally allows for analysis of the directional sound field. Finally, an anechoic sample of a snare drum hit was convolved with the binaural room impulse responses.

The SD (near 6 m, middle 13 m, far 20 m) and SL (left −15° azimuth, center 0°, right 32°) impulse responses were captured in a concert hall (1700 seats, reverberation time at mid frequencies 2.4 s). Thus, the rendered samples have a fair amount of reverberation. The impulse responses for small (class room), medium (smaller concert hall) and large (the same concert hall that was used for distance and location impulse responses) rooms were captured at different distances. Because of this SR sounds were adjusted in intensity so that the direct sounds were presented at an equal level, thus making sound sources apparently equal in direct-sound level (the primary distance cue) distance but differing in the effect of the room.

The nine binaural sounds (truncated to 500 ms) were then used to construct sound pairs (100 ms intervening gap) in which either the spatial distance, spatial location, or spatial room varied. For each stimulus condition, nine such pairs (duration of the pair 1100 ms) were generated, corresponding to all combinations of the three possible stimulus values (e.g., left, center, and right). Of the nine, three pairs presented no change and six presented a change. Depending on the task condition (see below), either the no-change pairs or all possible pairs were presented, in random sequence. Stimuli were presented within a continuous binaural background, in order to provide a reverberant spatial context for the sounds. The background sound (duration 18 s, ca. 30 dB below the intensity of SD, SL and SR sounds) was a “virtual symphony orchestra” playing Mozart using the impulse response of the same concert hall that was used to create the SD and SL sounds (Lokki et al., 2012).

Visual stimuli consisted of Gabor gratings (duration 100 ms). The orientation of Gabor gratings varied from −60 to 60° (in 14 steps).

The auditory stimuli were delivered binaurally using Sensimetrics S14 insert earphones (Sensimetrics Corporation, USA). The noise of the scanner (ca. 97 dB LAeq) was attenuated by the insert earphones, circumaural ear protectors (Bilsom Mach 1, Bacou-Dalloz Inc., USA) and viscous foam pads attached to the sides of the head coil. The sounds were presented at a moderate, comfortable listening level adjusted individually for each participant. The visual stimuli were presented in the middle of a screen viewed through a mirror fixed to the head coil. The experiment was controlled using the Presentation software (Neurobehavioral Systems, Albany, CA, USA).

The auditory stimuli were presented with natural spatial characteristics as would be present when listening to real sounds in space. However, we did not quantify the degree to which listeners perceived the stimuli as located within the head or “externalized.” Indeed, many characteristics of the sound presentation in this case are more consistent with the expectation of in-head than external perception (Toole, 1970). During the pre-fMRI training phase (see below), however, many of the subjects reported that they heard sounds as externalized.

### Tasks and sound sequences

During fMRI, subjects performed discrimination or 2-back tasks on sounds that varied in either of the three spatial dimensions, or performed a visual task in which they ignored the sounds and detected orientation changes in Gabor gratings.

The stimuli were presented in 18 s blocks alternating with 7 s breaks that contained no stimuli. During the breaks, subjects focused on a fixation mark (×) presented in the middle of a visual display and waited for the next task. A graphic task instruction symbol replaced the fixation mark 3 s before the onset of the next task block and remained on the screen until the end of the block. The graphic task instruction symbol indicated both the nature of the task (discrimination, 2-back or visual task) and the nature of the stimulus variation to base judgments on in the auditory tasks (SL, SD, or SR sounds).

In the discrimination task performed on SD sounds, subjects were to press a button with their right index finger when the two sounds were equal in distance (i.e., when they heard a no-change pair). Correspondingly, in the discrimination task performed on SL or SR sounds, subjects responded to pairs in which the sounds of the pair were identical on the corresponding dimension. In the 2-back task, only no-change pairs were presented. The subjects were required to press a button when the stimulus value was equal to the sound pair presented two trials before. In the visual task, subjects detected changes in the orientation of Gabor gratings. Sound sequences presented during the visual tasks were identical to the ones presented during the discrimination tasks. Visual stimulus sequences were presented also during the auditory tasks.

Each task block (duration 18 s) consisted of 12 sound pairs (pair onset-to-onset interval 1400–1600 ms, step 10 ms, rectangular distribution) and 51 Gabor gratings (duration 100 ms, onset-to-onset interval 150–350 ms). In addition, the background sound was played during each block. The purpose of this auditory background was to provide cues of a more natural acoustic space and to reduce the perceptual effects of scanner noise through partial masking. For each of the nine (2 auditory tasks with 3 auditory spatial dimensions + visual task with sounds from the 3 auditory discrimination tasks) different task conditions, there were 14 blocks resulting in (9 × 14) 126 blocks altogether. There were 2–4 targets in each block.

### Pre-fMRI training

Before fMRI, each subject was trained on all tasks (ca. 1 h) until they and study personnel felt confident in subjects' ability to properly identify the graphic task-instruction symbols and to correctly perform the corresponding (and demanding) tasks.

### Analysis of behavioral performance

Mean hit rates (HRs), reaction times (RTs), false alarm rates (FaRs), and d′ were calculated separately for each condition. Responses occurring between 200 and 1600 ms from the onset of the target stimulus were accepted as hits. Other responses (i.e., extra responses after a hit or responses outside the response window) were considered as false alarms. HR was defined as the number of hits divided by the number of targets. FaR was defined as the number of false alarms divided by the number of non-targets (i.e., change pairs). HRs and FaRs were used to compute the d′ [index of stimulus detectability, d′ = *z* (HR) − *z*(FaR)]. RTs were calculated only for hits. Behavioral results were analyzed using repeated measures ANOVAs and *t*-tests.

### fMRI data acquisition and analysis

fMRI imaging was performed on a 3.0 T MAGNETOM Skyra scanner (Siemens Healthcare, Erlangen, Germany) with a 20-channel head coil. Functional images were acquired using a gradient-echo echo-planar (GE-EPI) sequence (*TR* = 2070 ms, *TE* = 30 ms, flip angle 78°, voxel matrix 96 × 96, FOV = 18.9 cm, slice thickness 2.0 mm with no gap, in-plane resolution 2 mm × 2 mm, number of slices 27). The middle EPI slices were aligned along the Sylvian fissures based on high-resolution anatomical image (resolution 1.0 × 1.0 × 1.0 mm). The imaging area covered the superior temporal lobe, insula, and most of the inferior parietal lobe in both hemispheres.

Functional scanning was performed in two ca. 25 min runs resulting in 2 × 772 volumes. After the first run, there was a short break during which subjects remained in the scanner and were instructed not to move their heads or speak. After the functional scans, an anatomical image using the same imaging slices as in EPI but with denser in-plane resolution was acquired (voxel matrix 256 × 240).

Global voxel-wise analysis was performed using FSL (release 4.1, www.fmrib.ox.ac.uk/fsl). Data from the two runs were initially combined into one file for motion correction. The motion-corrected data were again split into two separate files, high-pass filtered (cutoff 100 s), and spatially smoothed (Gaussian kernel of 5 mm full-width half-maximum). Based on the timing information recorded during the experiment, each functional image was labeled as discrimination (performed on SD, SL or SR sounds), 2-back memory (SD, SL or SR), visual task (with sounds of the discrimination task using SD, SL or SR sounds), or baseline (7 s breaks containing no stimuli). The hemodynamic response function was modeled with a gamma function (mean lag 6 s, *SD* 3 s) and its temporal derivative. Finally, several contrasts were specified to create Z-statistic images testing for task and stimulus effects. A second level statistical analysis using fixed-effects combined the data from the two runs.

For analysis across participants (third level analysis), the high-resolution anatomical images were normalized in spherical standard space using FreeSurfer (release 5.1.0, http://surfer.nmr.mgh.harvard.edu). The anatomically normalized surfaces were rotated and projected to a two dimensional (2D) space separately for each hemisphere using equal area Mollweide projection (Python libraries matplotlib and basemap, http://matplotlib.sourceforge.net). This procedure was then applied separately for each subject to transform the results of the 3D second-level statistical analysis to 2D. Finally, the group analysis (FMRIB's local analysis of mixed effects, *N* = 17) was run on the flattened data. Z-statistic images were thresholded using clusters determined by *Z* > 2.3 and a (corrected) cluster significance threshold of *P* < 0.05 (using Gaussian random field theory).

### Regions-of-interest (ROIs)

Two anatomical ROIs (PT and IPL) were defined in the flattened 2D space for each hemisphere (see **Figure 2D**). One ROI was hand drawn to cover the PT of left hemisphere (following Figure 12.1 of Hickok and Saberi, 2012). The left hemisphere IPL ROI was based on the IPL cluster observed in our previous study during auditory tasks performed on spatially varying sounds (Rinne et al., 2012). The right hemisphere ROIs were then defined based on the left hemisphere ROIs.

## Results

### Performance during fMRI

**Figure 1:**
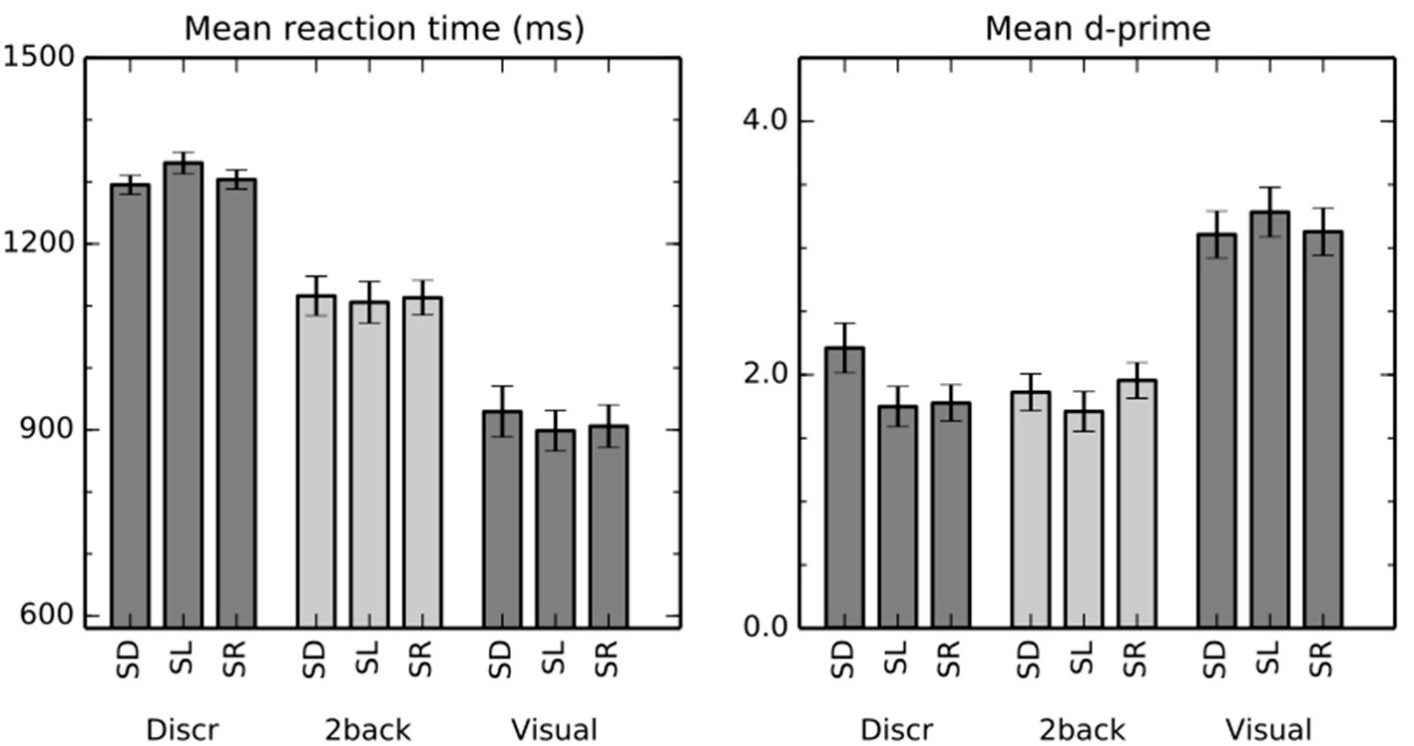
**Performance in the auditory and visual tasks**. The bars show mean (±s.e.m) reaction times and d′ in discrimination (Discr), 2-back (2-back), and visual (Visual) tasks with sounds varying in distance (spatial distance, SD), location in horizontal plane (spatial location, SL), or that were emitted in different rooms (spatial room, SR).

Mean reaction times (RT) and d′ are shown in Figure 1. Importantly, performance was similar across all auditory conditions. Nevertheless, some differences appeared statistically significant despite their small size.

A two-way repeated measures ANOVA with factors of task (discrimination, 2-back) and stimulus (SD, SL, SR) indicated a significant main effect of task on RT [*F*_(1,16)_ = 65, *P* < 0.001]. The difference is consistent with the fact that successful discrimination requires perception of both sounds in a pair, whereas the stimulus identity of the first sound could suffice in the 2-back task. There were no systematic differences between the stimulus conditions across the tasks [main effect of stimulus *F*_(2, 32)_ = 0.9, *P* = 0.4]. However, task × stimulus interaction was significant [*F*_(2,32)_ = 6.0, *P* < 0.01]. Specifically, in discrimination tasks, RT was slower when the task was performed on SL than on SD (*t* = 2.6, *P* < 0.05) or on SR (*t* = 2.6, *P* < 0.05) sounds, whereas there were no significant differences between the stimulus conditions in 2-back tasks.

Mean d′ was close to two in all auditory task conditions. The main effect of stimulus [*F*_(2, 32)_ = 4.9, *P* < 0.05] and the task × stimulus interaction [*F*_(2, 32)_ = 6.1, *P* < 0.01] were significant. In discrimination task, d′ was significantly lower for SL than SD sounds (*t* = 3.1, *P* < 0.01) and for SR than SD sounds (*t* = 3.4, *P* < 0.01). Other differences between the stimulus conditions were not significant.

### fMRI

**Figure 2:**
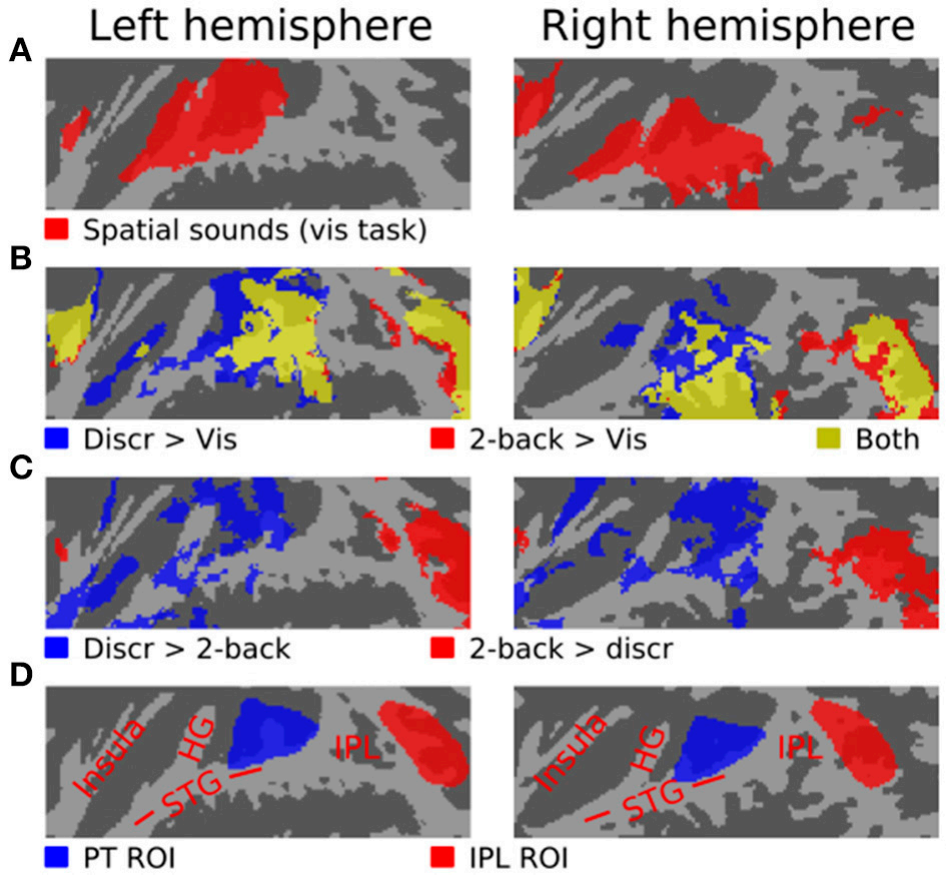
**Activations (*N* = 17, threshold *Z* > 2.3, cluster-corrected *P* < 0.05) to spatial sounds shown on a flattened mean 2D cortical surface. (A)** Results of contrast between activations during all visual task blocks (SD, SL or SR sounds) and during the 7 s periods between task blocks with no stimuli. **(B)** Comparisons of activations during all discrimination (blue) and all 2-back task blocks (red) relative to rest. Areas where both contrasts were significant are shown in yellow. **(C)** Areas where activations were stronger during discrimination than 2-back tasks are shown in blue. The results of the opposite contrast (i.e., stronger 2-back activations) are shown in red. **(D)** Anatomical labels and ROIs. STG superior temporal gyrus, HG Heschl's gyrus, IPL inferior parietal lobule.

As compared with the 7 s periods with no stimuli between the task blocks, spatial sounds presented during the visual task (i.e., in the absence of directed auditory attention) activated areas of anterior and posterior STG, insula and IPL (Figure 2A). During discrimination and 2-back task blocks, activations to sounds were enhanced in insula, posterior STG and IPL, and in discrimination tasks, also in anterior STG (b). As compared with each other, activations during discrimination task blocks were stronger in insula, anterior STG and posterior STG, whereas during 2-back blocks activations were enhanced in insula and IPL (c).

**Figure 3:**
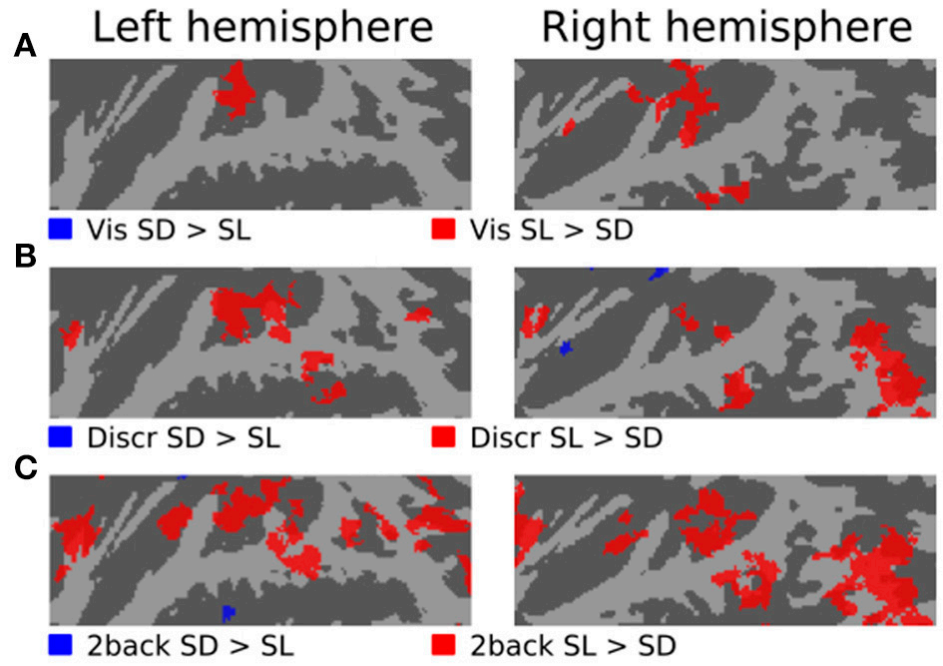
**Comparisons of activations during visual, discrimination and 2-back tasks with SD and SL sounds**.

**Figure 4:**
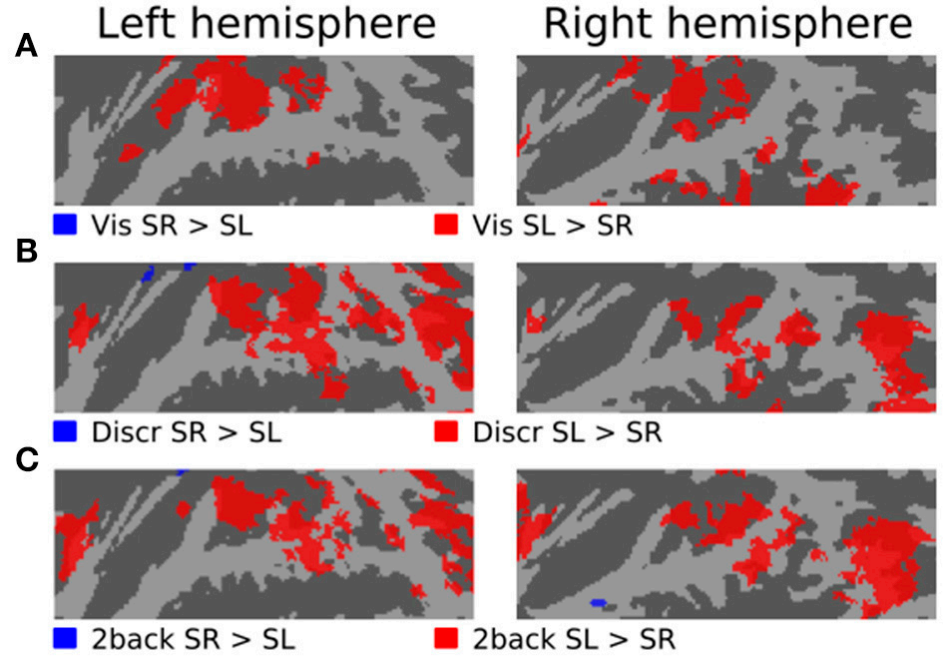
**Comparisons of activations during visual, discrimination and 2-back tasks with SR and SL sounds**.

**Figure 5:**
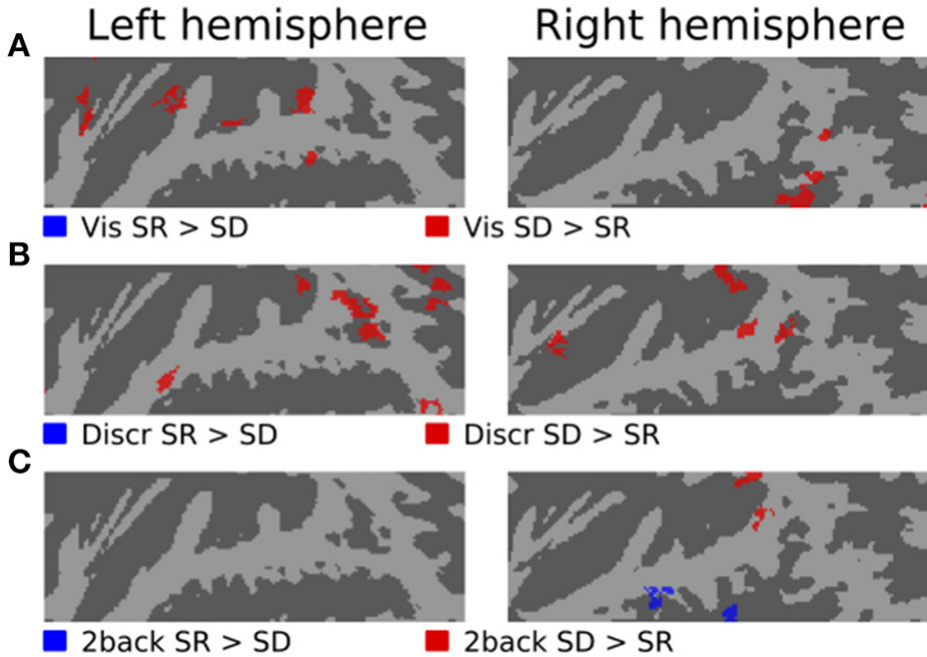
**Comparisons of activations during visual, discrimination and 2-back tasks with SR and SD sounds**.

Figures 3–5 show contrasts between SD, SL, and SR blocks presented during visual, discrimination and 2-back tasks. Activations in STG were stronger during SL than SD blocks during the visual task (Figure 3A). These activation enhancements were focused in PT bilaterally. During discrimination (b) and 2-back (c) tasks activation enhancements during SL blocks were detected, in addition to PT/STG, in insula and IPL. Similar patterns of activation enhancements associated with SL blocks were seen also in contrasts between SL and SR blocks (Figure 4). Contrasts between SD and SR blocks showed some scattered, less systematic activation enhancements associated with SR blocks (Figure 5).

### ROI analysis

**Figure 6:**
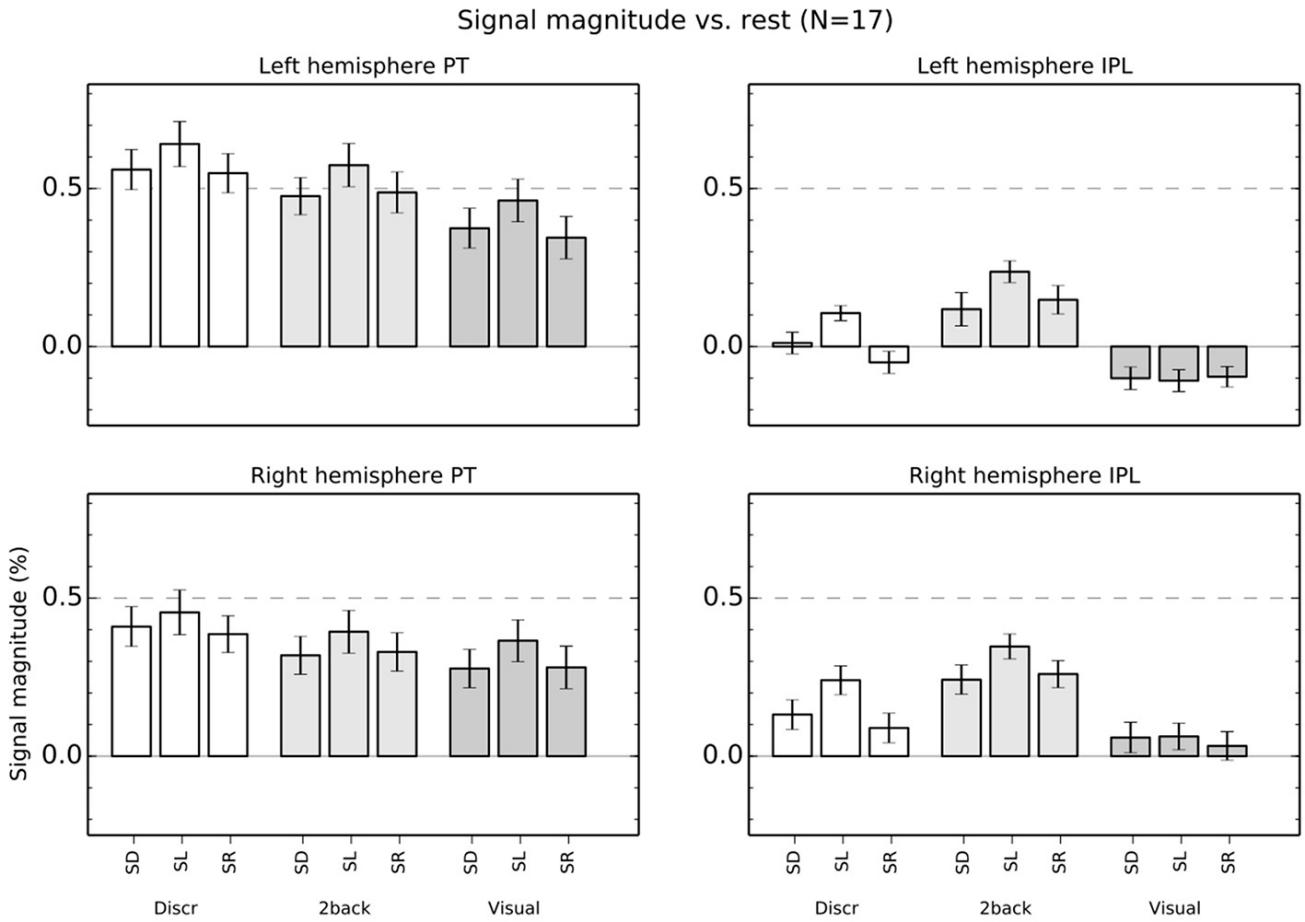
**Percentage signal magnitude in left and right hemisphere planum temporale (PT) and IPL ROIs (see Figure 2D)**. The bars show mean (±s.e.m.) ROI signal relative to rest.

ROI mean signal magnitudes (±s.e.m.) in each condition relative to rest (i.e., the 7 s breaks with no stimuli) are shown in Figure 6. An ANOVA for the left hemisphere ROIs with factors roi (PT, IPL), task (discrimination, 2-back, visual) and stimulus (SD, SL, SR) indicated significant main effects [roi *F*_(1,16)_ = 45, *P* < 0.001; task *F*_(2,32)_ = 37, *P* < 0.001; stim *F*_(2,32)_ = 27, *P* < 0.001]. Interactions roi × task [*F*_(2,32)_ = 18, *P* < 0.001], task × stim *F*_(4,64)_ = 2.7, *P* < 0.05] and roi × task × stim *F*_(4,64)_ = 8.8, *P* < 0.001] were also significant.

Subsequent ANOVAs conducted separately for both left hemisphere ROIs showed significant main effects in PT [task: *F*_(2,32)_ = 25, *P* < 0.001; stim: *F*_(2,32)_ = 25, *P* < 0.001] but no interaction [*F*_(4,64)_ = 0.7] and, in IPL, significant main effects [task: *F*_(2,32)_ = 34, *P* < 0.001; stim: *F*_(2,32)_ = 17, *P* < 0.001] and a task × stim interaction [*F*_(4, 68)_ = 6.1, *P* < 0.001]. This interaction in IPL was because activations during SD, SL, and SR sounds were different in discrimination [one factor ANOVA, *F*_(2,32)_ = 15, *P* < 0.001] and 2-back [*F*_(2, 32)_ = 7.7, *P* < 0.01] task but not during visual task [*F*_(2, 32)_ = 0.2].

Correspondingly, in the right hemisphere, the ANOVA roi × task × stim showed significant main effects [roi *F*_(1,16)_ = 7.6, *P* < 0.05; task *F*_(2,32)_ = 23, *P* < 0.001; stim *F*_(2,32)_ = 19, *P* < 0.001] and interactions [roi × task *F*_(2,32)_ = 20, *P* < 0.001; roi × task × stim *F*_(4,64)_ = 5.3, *P* < 0.01]. The ANOVAs conducted separately for each right hemisphere ROI revealed significant main effects [task: *F*_(2,32)_ = 13, *P* < 0.001; stim: *F*_(2,32)_ = 14, *P* < 0.001] but no interaction [*F*_(4,64)_ = 1.0] in PT, and significant main effects [task: *F*_(2,32)_ = 25, *P* < 0.001; stim: *F*_(2,32)_ = 17, *P* < 0.001] and a task × stim interaction [*F*_(4,64)_ = 3.8, *P* < 0.01] in IPL. As in the left hemisphere, this interaction in IPL was because activations during SD, SL, and SR sounds were different in discrimination [one factor ANOVA, *F*_(2,32)_ = 11, *P* < 0.001] and 2-back [*F*_(2, 32)_ = 8.9, *P* < 0.001] task but not during visual task [*F*_(2, 32)_ = 1.4].

## Discussion

### Activations to spatial sounds during discrimination, 2-back and visual tasks

As compared with each other, activations during auditory spatial discrimination tasks were enhanced in insula and STG (Figure 2C, blue), whereas during auditory spatial 2-back tasks enhanced activations were detected mainly in IPL (red). This activation difference between discrimination and 2-back tasks was similar irrespective of whether the tasks were performed on SD, SL, or SR sounds (not shown). Together with our previous studies of auditory discrimination and n-back tasks involving other acoustic dimensions, the present results strongly suggest that the main features of these task-related modulations reflect characteristics of the tasks and not the processing of stimulus-specific information (Rinne et al., 2009, 2012; Harinen and Rinne, 2013).

In the present study, activations during discrimination and 2-back tasks were also modulated by whether the task was performed on SD, SL, or SR sounds. In particular, activations were stronger during SL than SD or SR blocks in insula, STG and IPL (Figures 3B,C, 4B,C, 5B,C, red). These effects were partly due to stimulus-dependent processing as similar activation differences between SD, SL, and SR sounds were observed also during the visual task. However, during auditory tasks activation enhancements to SL sounds extended to wider areas in STG and insula. Notably, a distinct enhancement of IPL activations during SL blocks was detected only in auditory tasks (Figure 6). These results suggest that stimulus-specific information is processed similarly in areas of PT during both visual and auditory tasks but sensitivity to features of SL stimuli (such as binaural differences) expands to encompass additional regions in insula, posterior STG and IPL during auditory tasks.

### Activations in PT

In PT, activations were higher for SL than SR or SD sounds during auditory and visual tasks (Figure 6). These results are consistent with the large population of binaural azimuth-sensitive neurons in AC (Brugge et al., 1994; Harrington et al., 2008; Kitzes, 2008) and with previous studies and views implicating PT in processing of location of sounds in the horizontal plane (Warren and Griffiths, 2003; Barrett and Hall, 2006; Deouell et al., 2007; Rauschecker, 2011).

However, PT activations do not necessarily reflect processing of spatial information as such. Smith et al. (2010) compared PT activations to speech from one or three talkers presented from one or three locations. They found that PT responded more to spatially varying than non-varying stimuli, but observed similar PT activation increases when speech from three talkers (vs. one talker) was presented from one location. Based on these results, they argued that spatial sensitivity in PT might reflect the impact of spatial information on auditory stream segregation rather than the processing of spatial information as such. In the present study, pairs of drum sounds were presented in three different spatial conditions, each with three levels. In SL conditions, the pairs consisted of sounds from three horizontal locations (i.e., binaural cue values). In SD conditions, horizontal location was fixed but spatial distance (i.e., intensity and direct-to-reverberant ratio) varied in three steps. In SR conditions, horizontal location and distance were fixed but the sounds were emitted in three different rooms. Further, in all conditions the sounds were presented against a background sound consisting of an orchestra playing Mozart in a reverberant concert hall. Thus, it could be argued that the requirements for auditory stream segregation were equal in all conditions as the sounds had to be segregated from the background and there were three different sounds. Yet, SL conditions (varying horizontal location) resulted in stronger PT activations than SD and SR (fixed horizontal location in front) conditions, despite similar performance in all stimulus conditions (see, Figure 1). This result seems to suggest that PT is indeed more involved in processing of the spatial/binaural features of sounds. However, the enhanced PT activations to SL sounds could also reflect differences in the spatial segregation or grouping cues provided by SL sounds compared to SD and SR sounds.

In the present study, activations in PT were also modulated by tasks. PT activations to sounds were stronger during auditory than visual tasks and stronger during discrimination than 2-back tasks (Figures 2B,C, 6). In the discrimination task, the sounds varied both within the pair and between the pairs and subjects were to respond when the first and second part of a pair were identical. In 2-back task, the pairs contained two identical sounds and varied only between pairs. It is possible that higher PT activations during auditory than visual tasks were because the auditory tasks resulted in a higher load on stream segregation. Further, it is possible that the requirements for stream segregation were even higher during discrimination (both within and between pair variation) than during 2-back tasks (only between-pair variation). Thus, these PT activation patterns could be consistent with the view that PT is involved in auditory stream segregation.

We used measurements of real spaces to create spatial sounds. As a result, the SL sounds contained several different monaural and binaural cues for horizontal location. However, the main location cues (interaural level and time differences) required comparison of the signals in left and right ears. In contrast, differences between SD and SR sounds could be determined mainly based on monaural cues (intensity and direct-to-reverberation ratio) although all sounds were binaural with a sound source in front. As discussed above, PT activations were enhanced during SL blocks and there were no systematic PT activation differences between SD and SR blocks. In line with the previously proposed notion that PT acts as a “computational hub” (Griffiths and Warren, 2002), the present PT activations could be due to additional processing or greater salience of binaural cues, or due to greater coordination of information from the left and right hemifields required during SL but not in SD and SR blocks. Notably, Kopčo et al. (2012) demonstrated similarly localized PT activations for sounds varying in distance vs. intensity. Because the sounds in that study were synthesized for locations directly opposite the right ear at distances within 1 meter, the primary distance cue was provided by the interaural level difference (Brungart and Rabinowitz, 1999), and PT activations were mainly confined to the left (contralateral) hemisphere. The similar results of Kopčo et al. (2012) to those for SL but not SD blocks thus strongly suggests that enhanced PT activations reflect sensitivity to binaural differences *per se*, rather than distance processing.

### Activations in IPL

In the IPL ROI, activations during visual task did not significantly differ during presentation of SD, SL, and SR sounds (Figure 6). However, during auditory tasks, IPL activations showed enhanced activations to SL sounds similar to PT. These results suggest that IPL does not process physical information on the horizontal location of sounds as such and that during discrimination and 2-back tasks SL sensitivity in IPL may reflect further elaboration of inputs from PT.

In our previous studies using similar discrimination and 2-back tasks, IPL activation enhancements have been mainly observed in 2-back tasks. Some IPL activation enhancements were observed in a spatial discrimination task (Rinne et al., 2012), but not when the discrimination task was performed on pitch varying sounds (Rinne et al., 2009) or on vowels (Harinen and Rinne, 2013). However, in the present study, a distinct IPL activation enhancement was observed in both n-back and discrimination tasks (Figures 2B, 6). Enhanced IPL activations have been implicated with listening in adverse conditions and task difficulty (Obleser et al., 2007; Rinne et al., 2009; Leung and Alain, 2011). Thus, it is possible that the present discrimination tasks were more demanding than the discrimination tasks used in our previous studies. However, the present IPL activations associated with discrimination tasks cannot be easily explained with a general task difficulty effect. First, in the present study, performance (d′ in discrimination tasks performed on SD, SL, and SR sounds: 2.2, 1.7, 1.8) was in the same range as in our previous study with a spatial discrimination task (easy, medium and hard tasks: 1.8, 2.1, 1.6). Although one should be cautious in comparing performance in two separate studies conducted on different subjects, this suggests that the present discrimination task (with IPL activations) was not considerably more difficult than the discrimination task of our previous study (no IPL activations). Second, in our previous studies, we deliberately modulated task difficulty in pitch and spatial discrimination and n-back tasks. In those studies, increased discrimination task difficulty resulted in decreased performance but only weak effects of task difficulty on IPL activations were observed. In contrast, increased task difficulty in n-back tasks strongly enhanced IPL activations. Thus, any effects of discrimination difficulty on the present IPL activations would appear to arise not from the perceptual difference between the sounds to be discriminated, but from some other characteristics of the present task.

Alternatively, it is possible that the present discrimination tasks, especially during SL blocks, were associated with enhanced IPL activations because the tasks were performed on spatial sounds. Areas of posterior STG and IPL have been implicated in spatial processing (e.g., Alain et al., 2010) and, as mentioned above, some IPL activations were also detected in our previous study using a similar discrimination task with spatially varying sounds. However, the present IPL activations were stronger during 2-back tasks than during discrimination tasks. In our previous studies, strong IPL activations have been observed in n-back tasks performed on spatially fixed sounds (varying in e.g., pitch). Thus, IPL activations observed during both 2-back and discrimination tasks cannot be easily explained in terms of spatial processing on its own.

IPL has also been implicated in categorical processing (Chang et al., 2010; Leung and Alain, 2011; Harinen and Rinne, 2013). Our previous studies have demonstrated strong IPL activations during categorical n-back tasks. In these tasks, subjects were required to remember and compare stimulus categories (e.g., high, middle and low pitch in Rinne et al., 2009), with several different stimuli in each category. Although, in the present study, there were only three different stimuli in a block, it is likely that subjects performed the 2-back tasks using category labels (SL: left, middle, right; SD: near, middle, far; SR: small, medium, large) rather than based on continuous representations of physical stimulus information. Thus, the present IPL activations observed in the 2-back tasks could quite likely reflect operations, maintenance and storage of categorical information. It is possible that categorical representations were also used in the present discrimination tasks as there was only one stimulus in each of three categories. In the discrimination tasks of our previous studies, subjects were to discriminate multiple sounds within one category, so that a categorical discrimination strategy would be less useful. A better strategy in that case might have been to directly compare sounds based on lower-level stimulus representations. Previous studies (Durlach and Braida, 1969; Hafter et al., 1998) suggest that two processing modes are used in two-tone discrimination tasks depending on the characteristics of the task. A “sensory-trace mode” is used when the task requires explicit comparison of the two stimuli and a more efficient “context-coding mode” is used when classification or categorical processing can be used instead. Thus, the relatively strong IPL activations observed during the discrimination task could reflect that listeners were able to perform the discrimination task using a categorical processing mode. Consistent with this notion, no or weak IPL activations were observed in the discrimination tasks of our previous studies in which the sounds varied randomly in multiple steps, thwarting the use of category labels and forcing the task to be performed in “sensory-trace mode.”

An intriguing issue in the current results is the enhancement of IPL activation during tasks performed on SL sounds as compared with those during SD and SR blocks. As discussed above, areas of IPL (part of the “where” stream) could be specialized for spatial processing and these areas could be especially sensitive to SL. However, the present results indicate that IPL shows sensitivity to SL only during auditory tasks and, thus, it is probably not participating in processing of spatial stimulus-level information independent of task. During auditory tasks, IPL showed a similar pattern of activations to SD, SL, and SR sounds as PT (i.e., SL higher than SD and SR). This suggests that for task-relevant features, IPL may inherit PT representations for further processing. According to this idea, IPL is functionally connected with PT, but IPL is not necessarily participating in spatial auditory analysis as such. Rather, IPL would be involved in more general processing related, for example, to operations on categorical representations of task-relevant features.

### Conflict of interest statement

The authors declare that the research was conducted in the absence of any commercial or financial relationships that could be construed as a potential conflict of interest.
